# Neonatal Abstinence Syndrome: Twins Case Series

**DOI:** 10.3389/fped.2017.00242

**Published:** 2017-12-22

**Authors:** Rajesh Pandey, Narmada Pandey Sapkota, Deepak Kumar

**Affiliations:** ^1^Department of Pediatrics, University of Texas, Health Science Center at Houston, Houston, TX, United States; ^2^Cleveland State University, Cleveland, OH, United States; ^3^Department of Pediatrics, MetroHealth Medical Center, Cleveland, OH, United States

**Keywords:** neonatal abstinence syndrome, intrauterine opioid exposure, severity of neonatal abstinence syndrome, dizygotic twins, monozygotic twins, newborn, genetic, determinants of neonatal abstinence syndrome

## Abstract

**Background:**

Use or abuse of opioids and related drugs during pregnancy increases the risk of maternal and neonatal morbidities, including increased susceptibility to Neonatal Abstinence Syndrome (NAS). Severity of NAS is determined by both environmental and genetic factors, but the level of influence each one of them has in determining the severity of NAS is not fully understood. Since the incidence and severity of NAS vary a lot among susceptible infants, twin studies might give us valuable insights into understanding the relative roles of environmental and genetic factors at the onset of the disease and during its progression. Higher concordance of occurrence and severity of NAS in monozygotic twins compared to dizygotic twins would suggest a genetic role in the pathogenesis of NAS. However, comparable concordance suggests a non-genetic or an environmental basis. In this case series, we report neonatal outcomes including severity of NAS among monozygotic and dizygotic twins.

**Methods:**

A retrospective chart review was performed for all newborn twins who were at risk of developing NAS, were born in our institution between January 2006 and December 2014, and had a gestational age of 30 weeks or greater.

**Results:**

During the study period, we identified seven sets (total of 14 infants) of eligible twins, comprising six dizygotic and one monozygotic twins, from a total of 550 infants who were at risk of developing NAS. Among the seven sets of twins, two sets were concordant for severe NAS and required pharmacological management, three sets of twins were concordant in not having severe NAS and did not require pharmacological management, and the remaining two sets were discordant, where one of the twins required pharmacological treatment.

**Conclusion:**

Five of the seven sets of twins in our study exhibited concordance and two sets showed discordance in withdrawal severity. Larger studies may help in understanding the roles of genetic and environmental factors in determining the severity of NAS.

## Introduction

Maternal use, misuse, and abuse of prescription and nonprescription opioids and other illicit drugs, including heroin and cocaine, are increasing in the United States (1–3). With increasing drug use, risks of various maternal and neonatal morbidities such as preterm birth, low birth weight, possible congenital anomalies (4), neonatal abstinence syndrome (NAS), and adverse neurodevelopmental outcomes have increased (5, 6).

The incidence and severity of NAS have been variably reported to occur in 50–90% of infants following intrauterine exposure to drugs that are known to cause NAS (5, 6). Among the infants with severe NAS, there is a wide variation in the length of their pharmacological treatment, duration of hospital stay, need for intensive care, and other morbidities associated with NAS (5, 6), but what determines the variations in outcome is not completely known.

Twin studies are a powerful tool in understanding the roles of genetic and environmental factors in determining the incidence and severity of diseases. Incidence of twins is about one in eighty in spontaneous pregnancy, and incidence of NAS is estimated to be 3.4–5.6 per 1,000 births, resulting in about 21,000 infants being susceptible to NAS (2). Based on these data, we calculated that, annually, about 260 pairs of twins born in USA are susceptible to NAS. Twins can be dizygotic (non-identical) or monozygotic (identical). Dizygotic twins share about 50% of the genetic material while monozygotic twins share almost 100%. The composition of placenta and membrane—amnion and chorion—helps in determining whether twins are monozygotic or dizygotic. All dizygotic twins have a dichorionic-diamniotic membrane. Monozygotic twins can be dichorionic-diamniotic (about a third of monozygotic twins), monochorionic-diamniotic, or monochorionic-monoamniotic, depending on the time of splitting of the fertilized egg. If dichorionic-diamniotic twins are of the same gender, have the same blood group, and appear identical, they are usually designated as monozygotic twins in twin studies. Higher concordance of incidence of disease in monozygotic twins compared to dizygotic twins suggests a genetic basis of the disease (7).

We hypothesized that twin studies might give us valuable information in understanding the relative roles of environmental and genetic factors in the onset and progression of NAS where incidence and severity of disease vary widely among susceptible infants. If concordance of occurrence of NAS or severity of NAS is greater in monozygotic twins than in dizygotic twins, it would suggest a genetic basis of pathogenesis of NAS. However, lack of higher concordance among monozygotic twins compared to dizygotic twins would suggest non-genetic or environmental basis in pathogenesis of NAS. In our study, we have defined “infants at risk of NAS” as infants with intrauterine exposure to opioids and related substances. We have defined “severe NAS” as NAS requiring pharmacological management. Also, we have classified twins having concordance pattern of NAS if both or neither of the twins showed severe NAS, and discordance pattern of NAS if one of the twins, but not the other, exhibited severe NAS.

## Materials and Methods

We mined the database of MetroHealth Medical Center’s Mother and Child Dependency Program—a multidisciplinary treatment program for drug-dependent mothers and their infants—to identify all twins born at a gestational age (GA) of 30 weeks or greater, who were at risk of developing NAS, and were born between January 2006 and December 2015. This study was approved by the Institutional Review Board. We collected the maternal and infants’ demographics and other factors related to severity of NAS, including need and duration of pharmacological treatment, duration of hospitalization, mean NAS scores (modified Finnegan score) in the first, second, and third week of life, maximum NAS score, and cumulative dose of Morphine: the first-line medicine for treatment of NAS at MetroHealth. In demographics, we collected information including gender, birth weight, GA at birth, and types of twins: monozygotic or dizygotic. We also collected details about intrauterine exposure of the infants to methadone, buprenorphine, heroin, cocaine, marijuana, tobacco, and drugs used for treatment of underlying psychiatric conditions in the mother. Additionally, we collected information on the type of feeding: breast feeding or formula feeding. Amnionicity and chorionicity was determined based on obstetric ultrasound and, when necessary and available, infant’s blood group and gender were also used to designate zygosity of twins.

## Characteristics and Outcome of Seven Sets of Twins

### First Set of Twins

This set of diamniotic-dichorionic (dizygotic) twins was born at 38 weeks of GA to a 30-year-old Caucasian woman through emergency cesarean section for non-reassuring fetal heart tracing. She had a history of heroin use (both snorting and intravenous routes), cocaine use, and tobacco smoking, as well as a history of anxiety and depression, which were not treated. She was in the Methadone maintenance program, taking 45 mg methadone daily at the time of delivery. Her last reported illicit drug use was more than 4 months prior to delivery. She tested negative for Hepatitis B, Hepatitis C, and human immunodeficiency virus (HIV). Both twin A and B were male, with a birth weight of 2,775 g (blood group, A positive) and 2,830 g (blood group, O positive), respectively. Both infants were predominantly formula fed. Their median NAS scores in the first week were 3 and 2, respectively. Neither of the twins required pharmacological management for NAS, and both were discharged home on day 8.

### Second Set of Twins

Diamniotic-dichorionic (dizygotic) twins were born at 38 weeks of GA to a 34-year-old Caucasian woman by scheduled repeat cesarean section. The mother had a history of intravenous heroin and cocaine use, tobacco smoking, as well as a history of anxiety and depression, which were not treated. She was in the Methadone maintenance program, taking 45 mg methadone daily at the time of delivery. Her last reported illicit drug use was more than 4 weeks prior to delivery. She was tested positive for Hepatitis C but negative for HIV and Hepatitis B. Both twin A and B were male with a birth weight of 2,365 g and 2,025 g, respectively. The infants’ blood type was not available in their medical records. Both infants were predominantly formula fed. Their median NAS scores in the first week were 3 and 4, respectively. Neither of the twins required pharmacological management of NAS and both were discharged home on day 8.

### Third Set of Twins

This set of diamniotic-monochorionic (monozygotic) twins was born at 38 weeks of GA by vaginal delivery to a 21-year-old Caucasian woman. She had a history of intravenous heroin and cocaine use, tobacco smoking, as well as a history of anxiety and post-traumatic stress disorders, which were not treated. She was in the Buprenorphine maintenance program, taking 8 mg buprenorphine daily at the time of delivery. Her last reported use of illicit drugs was more than 3 weeks prior to delivery. She was Hepatitis B, Hepatitis C, and HIV negative. Both twin A and B were female, with a birth weight of 2,861 g and 2,658 g, respectively. Both infants had O positive blood group and were exclusively formula fed. Their median NAS scores in the first week were 5 and 5, with the highest scores of 10 and 13 for twin A and B, respectively; both required NICU admission. Both infants required pharmacological treatment for NAS for 12 and 26 days and were discharged on day 26 and 36, respectively.

### Fourth Set of Twins

This set of dichorionic-diamniotic (dizygotic) twins was born at 36 weeks of GA by vaginal delivery to a 29-year-old Caucasian woman. She had a history of heroin use (both snorting and intravenous routes), along with cocaine and marijuana, tobacco smoking, as well as a history of anxiety and post-traumatic stress disorder, which were not treated. She was in the Methadone maintenance program, taking 60 mg methadone daily at the time of delivery. Her last reported illicit drug use was more than 4 months prior to delivery. She was Hepatitis B, Hepatitis C, and HIV negative. Twin A was a female, with a birth weight of 2,121 g, and Twin B was a male, with a birth weight of 1,823 g. Both infants were exclusively formula fed. Twin A required pharmacological treatment for 9 days and twin B did not require any pharmacological treatment. Both infants were discharged home on day 19.

### Fifth Set of Twins

This set of dichorionic-diamniotic (dizygotic) twins was born at 37 weeks of GA by vaginal delivery to a 32-year-old Caucasian woman. She had a history of using heroin, cocaine, marijuana and tobacco smoking as well as a history of unspecified mental health illness, which was not treated. She was not in any opioids assisted program. Her last reported illicit drug use was 4 weeks prior to delivery. She was Hepatitis C infected, but tested negative for HIV and Hepatitis B. Twin A was a female, with a birth weight of 2,410 g, and twin B was a male, with a birth weight of 2,505 g. Both infants were exclusively formula fed. Their median NAS scores in the first week were 2 and 3 for twin A and B, respectively, and they stayed in the normal newborn nursery (NBS). Neither of the infants required any pharmacological treatment and both were discharged home on day 8.

### Sixth Set of Twins

This set of dichorionic-diamniotic (dizygotic) twins was born at 34 weeks of GA by vaginal delivery to a 27-year-old Caucasian woman. She had a history of using heroin (by snorting and intravenous route), cocaine, marijuana as well as tobacco smoking, but no history of mental health illnesses. She was in the Methadone maintenance program and was taking 120 mg methadone daily at the time of delivery. She tested negative for Hepatitis B, Hepatitis C, and HIV. Twin A was a female, with birth weight of 2,121 g, and twin B was a male, with birth weight of 2,105 g. Both infants were exclusively formula fed and both required pharmacological treatment for withdrawals for 32 days. Twin A was discharged on day 43, while twin B was discharged on day 41.

### Seventh Set of Twins

This set of dichorionic-diamniotic (dizygotic) twins was born at 31 weeks of GA by vaginal delivery to a 31-year-old Caucasian woman. She had a history of heroin, cocaine, other opioids’ (not specified in chart) abuse and tobacco smoking, as well as a history of anxiety and depression, which were not treated. She was in the Methadone maintenance program and was taking 50 mg methadone daily at the time of delivery. She was tested negative for Hepatitis B, Hepatitis C, and HIV. Twin A was a female, with a birth weight of 1,440 g (blood group, A positive) and twin B was also a female, with a birth weight of 1,570 g (blood group, O positive). Both infants were exclusively formula fed. Twin A required 14 days of pharmacological treatment, whereas no pharmacological treatment was required for twin B. Both infants were discharged home on day 49. Severe NAS is not very common among preterm infants born at 31 weeks, with a birth weight of about 1,400 g. We thus looked for alternative diagnosis that could explain the need of morphine but could not find anything other than NAS.

## Discussion

Of the seven sets of twins, there was concordance of severity of NAS in five sets of twin siblings, which included a set of monozygotic twins, and discordance in two sets of twin siblings. Among the five pairs of twins with concordance of withdrawal severity, two pairs of twins developed severe NAS and three pairs did not develop severe NAS. Although the pair of identical twin siblings developed severe NAS and required pharmacological treatment, the length of treatment and hospital stay varied greatly among the twin siblings. Twin A was treated pharmacologically for 12 days and stayed in hospital for 26 days while B was treated for 26 days and stayed in hospital for 36 days. In the two pairs of dizygotic twins with discordance of NAS severity, one pair required medical treatment while the other did not. A total of 6 out of the 14 (42%) infants required treatment.

To the best of our knowledge, this is the first case series of twins with NAS. Among all twins, we found 70% concordance of severity for withdrawal as measured by requirement of pharmacological treatment and 30% discordance of severity. Adequately powered twin studies may help in delineating the influence of genetic and environmental factors in determining the severity of NAS.

## Author Contributions

All authors were involved in conceptualizing and designing the work, RP prepared the initial draft and all authors revised the initial draft and agreed on the final version as submitted.

## Conflict of Interest Statement

The authors declare that the research was conducted in the absence of any commercial or financial relationships that could be construed as a potential conflict of interest.
